# Book Reviews

**Published:** 1814-10

**Authors:** 


### CRITICAL ANALYSIS

### OF RECENT PUBLICATIONS

IN THE

### DIFFERENT BRANCHES OF PHYSIC, SURGERY, AND MEDICAL PHILOSOPHY.

---

*Review of some Observations on Medical Reform, made in the Pamphleteer No.* VI. *by a Member of the University of Oxford*.

THE sixth number of the Pamphleteer contains an anonymous pamphlet on Medical Reform, by a Member of the University of Oxford, and, if we are not mistaken, by a Fellow of the College of Physicians of London, from the zealous support he gives to that body, and the increased power he demands for them. He is a *seeming* enemy to reform whether political or private, but at the end of his production recommends one of the most despotic description that has ever yet been devised by the most strenuous advocate for medical reformation.

The object of this publication is to decry Edinburgh graduates, and exalt those of Oxford and Cambridge; and why? because, forsooth, at Oxford and Cambridge "they regularly attend prayers in the public chapel, and meals in the public hall, and lectures in the public room; never sleep out of their College, or return late within its walls." Thus it is that the Oxford doctors pray, eat, and sleep their way to their degrees, by keeping a certain number of terms, as barristers find their road to the bar; but that they undergo no examination, no real test of the sufficiency of their acquirements, is universally notorious.

We are willing to speak of those Universities with due respect, as venerable and respectable seminaries of classical and mathematical learning; but we affirm that they have no pretensions to the character of medical schools, for it is well known that they have no medical lectures which deserve the name, and the pupils are not obliged to be present even at the ceremony of their delivery, and may absent themselves without hearing the voice of admonition, or feeling the hand of controul. The professorship of physic at Cambridge is a sinecure, and no lectures whatever are delivered; and Sir Busic Harwood's "anatomical lectures are only calculated to convey general instruction, and are not confined to any particular profession."* The lectures on anatomy continue only a few weeks, and the Professor rarely has a fresh subject, unless the body of some unhappy criminal be remitted to him for dissection by the sheriff of the county. Their lectures, then, are superficial, and chiefly on comparative anatomy, and teach no more than every well-educated person ought to know. Clinical lectures are not known, nor do the students visit the sick in the hospital.

Woe, then, to the patients who fall into the hands of physicians thus instructed! Will it be urged that English doctors are often well educated, and men of celebrity in their profession? We grant it; but they obtain their knowledge at Edinburgh or London, where all of them go, not only to learn the elements of their profession, for that even Oxford and Cambridge cannot teach them, but to finish their medical education, and at these places some of them have laid the foundation of future and deserved eminence. What a figure would our Oxford doctor cut with a scalpel in his hand, if educated only at Oxford! Whence (if he has any) did he derive his knowledge of surgery, or the treatment of diseases? Surely not at Oxford. There are no dissecting-rooms at either of our English universities; and, if a fresh subject were to be exhibited, half the students of the place, whether of law, divinity, or general literature, would crowd to behold it; whereas at Edinburgh there are public dissecting-rooms in the college, under the superintendance of Dr. Monro and Mr. Fyfe, and several private ones in the town, conducted by anatomists of known talents.

Our author says, "in this course of study they continue four

---

* See Cambridge University Calendar.

years before the first degree can be taken, and the students of medicine must devote themselves to their particular studies for three years more, before they can receive another degree, which only entitles them to apply for a licence to practise or become physicians. If the physician aspires to be a doctor of medicine, he must wait four years longer, before he is entitled to receive this honour." All this we admit; but let us ask for what good purpose? Is it not well known that a mere term-trotter, who attends "prayers in the public chapel, and meals in the public hall," for a few weeks in the year, and spends the rest of his time in snipe-shooting, or any other idle amusement, is entitled to the same privileges, without even the ceremony of an examination, or trial that deserves that name, and without the possibility of obtaining any professional information? And this is the education to be obtained in the "English universities, which will place the men who are fortunate enough to be educated in them above the level of ordinary men."—"Physicians," we are told, "rank as barristers and clergymen; but the English universities, when they confer the degree of doctor, elevate a man to a rank next to a knight." The self-importance of our author is really ludicrous; let him beware, lest, like the little animal in the fable, his intumescence be the cause of accident.

And again: "At the school of Edinburgh (for it is miscalled an university) the teaching is elementary." What, then, have the celebrated Cullen, the Monros, the Blacks, the Gregorys, the Homes, and many others, frittered away their time in teaching only the elements of their profession? Where, then, did Hunter, Currie, Darwin, Brown, Percival, Fothergill, Saunders, with a host of the most eminent physicians of the present day, obtain that information which elevated them to the highest pinnacle of professional fame, for they were not "fortunate enough to be educated at an English university?" Let us look around us, and we shall see an Edinburgh graduate, not "pushing the old apothecary out of his place, and becoming the middle man," but taking the lead in practice in almost every town in the kingdom, not because his degree gives him any particular privileges, but because he has earned that situation, and is entitled to it by his professional acquirements.

Three years study our author thinks too short to be admitted to practice; and we agree with him that it is, and should be glad to see the time extended to four or five years; but we doubt whether there are more raw boys sent from the Scottish universities, in proportion to their numbers, than from the English, where very few medical men are educated; and we know instances of young men from the latter being, on account of their youth, refused admittance into a sick room, or their prescription put into the fire when they have been called in by mistake, or sent in lieu of another. It is again objected, that "at Edinburgh there is no academical distinction of dress, the student is placed under no restraints, *he attends no chapel, no hall,* no lecture even unless he pleases; even in the slippery period of early youth he is exposed to every temptation, and very often sinks to the lowest debauchery."

Where

Where there are many hundred young men assembled together, it is probable some may give loose to vicious habits; but the punctual attention at lectures, the sober and orderly behaviour of the students at Edinburgh is remarkable, and universally acknowledged. Are there no debauchees in the English universities? Is it not allowed, that half of the young men who enter them learn nothing, and forget the little they brought with them from school? Was not their lax discipline and deficient acquirement so notorious, so crying an evil, as within these few years to demand more strict examinations, and new regulations? Is not the chapel considered, in the language of Oxford, "a bore," where the young men cut their nails, and applaud the scholar who can get through the service soonest? Are Oxford graduates more regular than others in their attention on divine service, when they sit down to practise their profession? Are there no instances of general riot and insubordination there? Their discipline, we allow, is adapted to the education of priests, but not of physicians. Were it not invidious, we could point out as many instances of debauched and tippling M. Doctors of Oxford manufacture, notwithstanding the pure and celibate life their fellowships inculcate, as issue from the Scottish universities. When our "monk of Magdalen" calls our attention to the devotion of the public chapel, and temperance of the public hall, he forgets the riot of the coffee-house, and the dull and deep potations of the common room. Let our author be informed, that, though they are not subjected to the gloomy and monkish sequestration of the cloister, nor encumbered with the pomp of a distinguishing dress, they may have as much morality, as much learning, as much professional knowledge, as the members of English universities, and undergo a more severe test of the real acquirement of that knowledge before a degree is conferred, than any other university in Europe.

"The new improvements," says the immortal Gibbon, speaking of English universities, "so eagerly grasped by the competition of freedom, are admitted with slow and sullen reluctance into those proud corporations, above the fear of a rival, and below the confession of an error; we may scarcely hope that any reformation will be a voluntary act, and so deeply are they rooted in law and prejudice, that even the omnipotence of parliament would shrink from inquiry into the state of abuses of the two universities."—"The greater part of the professors," says Dr. Adam Smith, "have given up even the pretence of teaching, being secure in the enjoyment of a fixed stipend, without the necessity of labour, or the apprehension of controul." We agree with our author in some particulars, viz. the necessity of distinction of ranks, and division of labour in medicine, and the impropriety of blending, as the levelling reformers would, the several duties of physician, surgeon, and apothecary; the functions of each ought to be performed by distinct classes of individuals; and it is the interest of the profession and the public that they should be: but we should be sorry to see the sovereignty over medicine placed in the hands of the College of Physicians and English doctors, according to the plan proposed; which is, "that three fellows lows of the College of Physicians shall form a *quorum;* or when three cannot be assembled, one fellow and two M.D.s of Oxford or Cambridge shall have the power of granting licences to practise; and none but English graduates be allowed to practise without them; that, north of the Tweed, and for the Colonies, Edinburgh and Glasgow grant licences to practise; that apothecaries be licenced and examined by members of the College or Society of Apothecaries, paying a licence fee proportionate to the population of their place of residence; that druggists be sub-licenced; that pharmaceutical poisons be laid under strict embargo, &c. &c. Of part of this plan we must approve; but, since the act of union between the two countries gives "a communication of rights, privileges, and advantages, which do belong to the subjects of either kingdom,"* we will not consent that that degree which gives, and always has given to those who are possessed of it, the privilege of practising or teaching medicine, "ubique gentium," shall submit to the regulation of the doctors of any other university.

---

*Elements of Medical Jurisprudence; or a succinct and compendious Description of such Tokens in the Human Body as are requisite to determine the Judgment of a Coroner, and Courts of Law, in Cases of Divorce, Rape, Murder, &c. To which are added, Directions for preserving the Public Health.* By SAMUEL FARR, M.D. 12mo. pp. 139. 1814.

THE title of this little volume sufficiently proclaims its object: and we hope its appearance may excite in the practitioner an attention to a most important and much-neglected study. Whether we look upon the science of medical jurisprudence in its connection with the actual practice of medicine, or as one of the accomplishments of medical education, without which it cannot be said to be complete; it will be found highly deserving of our regard; but in the former point of view alone, it is a duty imperative on the practitioner to make himself acquainted with it to its fullest extent. The physician or surgeon is often called upon to make depositions in courts of justice respecting the cause of death under particular circumstances: should he err on one side, the existence of a fellow-creature may be forfeited through his ignorance; by a mistake on the other, the guilty may be absolved, and the purposes of justice defeated. How mortifying the situation of the practitioner, who, called as a witness on a trial, is unable to explain what is required of him, or delivers his evidence contrary to the dictates of science! Exposed to the buffetings of hostile counsel, his stupidity is soon brought to light; he retires an object of derision with the whole court, with a stain upon his character that the grave will scarcely efface! Setting aside, however, the personal interest we have in an intimate acquaintance with this science, can no case arise in which life may be endangered

* See fourth article of the Act of Union between England and Scotland.

by our ignorance of it? We fear many such instances could be found: one occurred within our own recollection, where an individual narrowly escaped being hanged upon the testimony of *several* medical men of eminence in Liverpool; and certainly would have paid the forfeit of his life, had it not been for the providential and unexpected interference of Dr. Carson, of the same place.

As no arguments we can adduce can have so much weight as this solitary fact, we shall call the attention of our readers to the particulars of the case; to which we shall subjoin another, which occurred in our own private practice, which strikingly proves the correctness of Dr. Carson's reasoning upon this subject.

A Mr. Angus, of Liverpool, was indicted for the murder of Miss Burns, a young woman with whom he lived in habits of stricter intimacy than prudence or modesty could justify. She became pregnant, miscarried, and died in a few days. The concealment of the delivery, and subsequent death, induced a suspicion that abortion had been produced by means of poison: and unhappily, on examining the body, *an opening or supposed ulceration was discovered in the posterior part of the stomach,* which was pronounced by gentlemen of eminence to be capable of explanation in no way but on the supposition of poison having been administered. Marks of inflammation were seen on the peritoneum, small intestines, *and also on the peritoneal covering of the uterus;* which last, being of the size of a bullock's heart, left no doubt of the very recent delivery of the deceased. By some unlucky fatality, the inflammation of the uterus excited no suspicion that all the mischiefs which terminated in death had there begun; upon the evidence of these gentlemen the unfortunate man was committed to goal, on suspicion of being the perpetrator of this atrocious deed; and, after an imprisonment of six months, was arraigned for the murder. When the trial came on, it should be mentioned, to the honour of Dr. Carson, that he alone stood forward and maintained, in opposition to his brethren, that the appearance of the stomach could be accounted for by the known fact of the occasional digestion of that viscus by the action of the gastric juice after death. And to this conflicting testimony of men of equal reputation, Mr. Angus was indebted for the preservation of his existence.

We should be sorry that any remarks of ours could merit the imputation of personality; but we are constrained to observe, that the evidence, levelled at this unfortunate man's life, was given in opposition to facts which ought to have been notorious to every one. If the instances of digested stomach, related by John Hunter,* were in the estimation of these mistaken witnesses not sufficiently analogous with the appearances of that viscus in the case of Miss Burns to warrant the entire exculpation of the prisoner, in a matter of such serious importance, the *great* uncertainty of all medical reasoning should have induced them to *admit that uncertainty* in giving their testi-

* This subject has been admirably illustrated by Dr. Adams, in his Essay on Morbid Poisons.

mony mony against him. Each party used Mr. Hunter's words to prove the fallacy of the other's reasoning; but neither seemed perfectly to understand him. Dr. Carson, by continually keeping alive the question concerning the stomach, contrived to keep that of the uterus out of sight. But his opponents were so little prepared for his objections, that they never could rally with sufficient firmness to direct the whole attention of the court to the state of the uterus.*

Without examining the question of the prisoner's criminality in having procured the abortion, we cannot entertain a doubt that the supposed ulceration of the stomach could not have been produced by the direct action of poison. If from future observation a connexion between inflammation of the abdominal viscera and the digested state of the stomach be shewn to exist, it may then become a consideration whether the inflammatory symptoms were produced by the operation of poison, or the delivery itself, under all the disadvantages attendant on its concealment. That the ulceration was quite independent of poison, we think will be satisfactorily proved by the following case.†

Thyrsa Binden, aged twenty-six, residing at No. 43, Great St. Andrew-street, was delivered of a male child, after a natural labour of six hours. Until the fifth day of her confinement she was as well as under ordinary circumstances, when she began to complain of pains in the abdomen. A neighbouring apothecary was employed, who considered the symptoms to be common colic; but thought so lightly of them, that he merely ordered a common fomentation, and gave a powder, supposed to be an antimonial preparation. On the third day from this attack, the pains having increased to an insupportable degree, another apothecary was sent for, who recommended an accoucheur to be consulted, when Mr. Clarke was called in, who ordered a large quantity of blood to be drawn from the arm. It was immediately done. In the evening the symptoms were evidently increasing, though at first relieved by the bleeding. We were now sent for. She complained of the most excruciating pain of the abdomen, with inability to sit in an erect posture. The surface of the body was so exquisitely tender, that the slightest pressure could not be borne, nor could she move herself without the most alarming increase of her sufferings. Her skin was hot, she had intense thirst, the pulse was full, quick, and undulating, and the tongue was covered with a whitish fur. She had taken a solution of sulphate of magnesia in mint-water, which had procured the necessary evacuations from the bowels.

Here was no time to be lost; and, well knowing the liberality of our predecessor, Mr. Clarke, we ventured to wave the etiquette of the profession, which made it proper for us to consult with him, previous to prescribing in this case. Thirty ounces of blood were or-

* For a most important investigation of this case, see our 21st volume, page 336.

† Mr. Want thinks it proper to acknowledge himself to be the writer of this article, that the public may judge how far the authenticity of the case may be relied on.

dered dered to be taken, and the abdomen to be kept constantly wet with cloths dipped in vinegar and water. No internal medicine was administered. In the early part of the night she was relieved; the lotion appeared to be serviceable while applied, but, from an unlucky though natural fear of checking the discharges which are peculiar to the puerperal state, they were laid aside, and no permanent benefit was experienced. The symptoms recurred in the night with their usual violence: on the following morning the bleeding was repeated, and the nurse being convinced of the necessity of re-applying the lotion, it was done. About noon she was evidently relieved. Mr. Clarke met us in consultation, and it was agreed to continue our present plan. In the evening she was free from pain; the lotion had been kept constantly applied; she spoke of it as being peculiarly grateful to her, and believed it in a great degree to have been instrumental in procuring the ease she experienced. She was now sanguine in her expectations of recovery; but there was a fixedness and anxiety in her countenance which left us little to hope. The breathing became so laborious as to render it necessary to support her in an erect position by pillows. In the morning the pulse became weaker; convulsive twitches of the tendons supervened; she was evidently sinking, and in the evening she died.

It should have been remarked, that no vomiting had taken place during any part of her illness. With some little difficulty we obtained permission to inspect the body after death. On dissection, marks of high inflammation were evident in the peritoneum and the external coats of the intestines, which were slightly connected together by a gelatinous substance, but were easily separable; the surface of the intestines was covered by pus, a large quantity of which was found in flakes floating in about a pint of fluid effused into the cavity of the abdomen; water was found in the chest and pericardium; but the appearances most important for our purpose, were those which presented themselves in the stomach: *the posterior part of that viscus was completely eroded, and the contents effused into the abdomen immediately behind it.* Patches of inflammation were also found both on its internal and external surfaces. The uterus was inflamed, and had not yet contracted to its natural size.

The slightest comparison of this case with that of Miss Burns, as related in evidence upon the trial, will abundantly satisfy the reader of their being as similar as two cases in different constitutions of body can well be supposed to be; both young women, dying within a few days after delivery, with symptoms of peritoneal inflammation, and both with *digested stomachs after death.**

In one respect only they differ, in our case, which was open to the inspection of the whole world, no possible suspicion of poison could

* These may be regarded as very important facts in pathology, and may lead us more closely to investigate the seeming connection between the inflammatory symptoms and the state of the stomach. But we dare not dwell upon this, as it is foreign to our present subject.

exist. In the other, probably the concealment of the delivery gave rise to the suspicion. Let us hence learn the danger of stepping aside from the abstract question presented to our decision, with the hope of obtaining assistance from collateral evidence, which is at best to be suspected, and certainly ought never to influence the judgment of the practitioner.

With regard to the merit of the publication before us, although it is calculated to impart much instruction to the uninformed reader, we cannot allow it to possess that degree of perfection which the present state of science would entitle us to expect; in many places it contains sentiments evidently erroneous, especially where the grounds of divorce are discussed; and the important question of the digestion of the stomach is left entirely unnoticed. We are happy to be enabled to add, that some recent arrangements at the Windmill-street Theatre may be the means of directing the attention of students in medicine to the study of this interesting science, as Dr. Richard Harrison has announced his intention of delivering a full course of lectures upon it.

The contents of this Epitome are arranged under nine heads:—Pregnancy—Parturition—Divorces—Rape—Murder of Infants—Homicide—Idiotism and Insanity—Impostors—Means of preserving Public Health. As the work is in itself much condensed, and scarcely capable of abbreviation, we shall, for the purpose of shewing the nature of its execution, make some selections from three of these chapters.

" When the word parturition relates to the child itself, it may denote the time when it is born, the conformation of its parts, or the external figure which it presents, the state of its life, and the number which are brought into the world.

" When it relates to the time in which it is born, it may be considered either as perfect or mature, or immature and imperfect. The former, when gestation has been carried on at least nine months: the latter, when it is completed before that time; and in this last case, another division may be made into abortions, where the delivery is made before the seventh month; and premature births, where the child is born between the seventh and the end of the ninth. To this head also belong too late deliveries.

" The signs of an immature child are taken from the following particulars:

" 1st. From its length, for, if it be not one foot long, we may be nearly certain that it is not completely formed.

" 2d. From its weight, which should exceed five pounds.

" 3d. From the figure of the head, &c. An incomplete child has a deformed face resembling an old person, with a wide mouth and slender ears like membranes; its eyes are shut; the hair of its head is of a whitish cast; the division between the bones of the skull, called the rhomboidal suture, gapes wide; the bones themselves are moveable; and the lips of the mouth resemble pieces of bloody flesh.

" 4th.

"4th. From its habit of body, which is for the most part thin and tender, and covered with a short down, and is of a reddish hue, particularly on the extremities and the face. If it be a male, the scrotum is of a round figure, and the testicles are not contained in it.

"5th. From its limbs, which are thin and weak, and the nails upon its fingers are soft, short, not extending beyond the fingers; nay, if it be very small, as of one or two months, the nails are by no means perceptible either upon the fingers or the toes.

"6th. From the conformation or constitution of its bones; for it is evident from experience, that in every month of gestation there is some alteration in this respect; ex. gr. in a fœtus of five months, the orbits of the eyes are entirely formed into bony sockets; and in one of seven months, the small bones, subservient to the organ of hearing are so perfect, as scarcely to differ from those of a complete child.

"7th. From the umbilical cord, which is very slender.

"8th. From other curious circumstances which attend this little embryo, such as a constant indulgence in sleep, an abstaining from crying, an intolerance of cold, an indisposition to suck, or to use its limbs, or the muscles of other parts, such as those which are subservient to the evacuation of urine, or the depositing of the meconium.

"The signs by which we distinguish a perfect child are taken,

"1st. From its size; its length being at least one foot six inches.

"2d. From its weight, which should be at least six pounds.

"3d. From the formation of its bones, which is known only by experience. But in general, a child can hardly be called complete, all whose bones, and every part are not entirely formed, though age may give some addition to their substance.

"4th. From the umbilical cord, which is thick and firm.

"5th. From other circumstances, opposite to those in that which was imperfect; such as that he cries, moves his limbs, opens his eyes, sucks at the breast, is not always asleep, can bear cold, has a white skin, can evacuate urine and the fæces, has long nails, and his head covered with hair.

"That which relates to the conformation of a child, after it is brought into the world, is distinguished into monstrous and not monstrous: the former including all deviations from the ordinary figure of man. Monsters are again divided into perfect and imperfect. A perfect monster is that which absolutely differs in all its parts, from the human appearance, as when it resembles any brute animals, as a dog, an ape, &c. An imperfect monster is where only a partial alteration is made in its figure; and this may again differ, according as this partial alteration is made in the head or other parts; and this as it may be born without a head, or with the head of a beast, &c. Where a monster differs from a complete child, in other parts besides the head, it is distinguished into two sorts, as any parts in general are affected, or as more particularly the change is wrought in the genitals only, and then it is called an hermaphrodite, which is likewise perfect or imperfect.

"In an enquiry into the nature of monsters in general, three objects of consideration present themselves. 1st. What is the cause of monsters? 2d. Whether are they possessed of life? 3d. Whether a perfect monster can be considered as a human being?

"1st. The cause of monsters is various, as depending on such changes in the constitution of the mother as can hardly be accounted for.

"Whatever view we take of the theory of generation, whether a germen be formed in the ovarium of the female, which is only impregnated by the semen of the male, or whether the homunculus is contained in that semen, and the female affords a nidus for its formation; still we see a strong resemblance to both parents in their offspring: and accidents, or other causes, contribute to make an entire alteration in the form of the fœtus, and produce monsters. We will not suppose unnatural connections, or that any impregnations can arise from that source; but imagination has a great power over the body of a female, especially during gestation; and the fluid in which the fœtus swims, or the womb itself may be disordered, so as to occasion great changes. Neither need we have recourse to the theory of the ingenious Buffon to explain how these are brought about; or suppose that every part of the human body has a representation in the fecundating quality of both parents, to form its construction. The first rudiments or germen of the human body is not a human creature, if it be even a living one; it is a foundation only upon which the human superstructure is raised. This is evident to anatomical observation. Were a child to be born of the shape which it presents in its first stages of pregnancy, it would be a monster indeed, as great as any which was ever brought to light. How easy then is it for disorder to prevent the exertion of that plastic force, which is necessary to form a complete animal.

"2d. Monsters may live, but it depends on what parts are affected, how long life shall be continued to them. Where the monstrous parts are confined to the extremities, or even to those places which distinguish hermaphrodites, we find from experience, that the vital powers are strong and vigorous; and were it not that such beings often fly from society, lead sedentary lives, and are deprived of some wholesome exercises to the human constitution, life might be enjoyed by them, and to as great an extent as by any other persons.

"3d. With regard to perfect monsters, most of the authorities which assert that any thing of that kind can exist, seem to be of no credit. But should any ever appear, we should consider that it is not form or shape, but reason and intelligence, which distinguish human creatures from brute animals.

"We are next to consider the nature of hermaphrodites; and as these are living beings, and sometimes capable of all the functions of society, such distinctions ought to be made relating to them, as will place their situation in the most proper light, and the most favourable to their happiness. They are great objects of our pity and complacency; for they are not only deprived of the common pleasures of mankind, but are subject to disorders which are painful, uncomfortable, fortable, and inconvenient. A perfect hermaphrodite, or a being partaking of the distinguishing marks of both sexes, with a power of enjoyment from each, is not believed by any one ever to have existed. Imperfect hermaphrodites, or monsters, whose organs of generation are affected, are frequently presented to us. They may be divided, according to the sexes, into what are called androgynus, and androgyna. The first is the male, who has in general his own organs tolerably perfect, but has some division in the flesh above, below, on, or in the scrotum, which puts on the appearance of the female pudendum. The penis likewise may be so obliterated, as to give no external appearance of the male; but the beard, and the constitution of his body, confirm him to be of that sex. The androgyna is a woman, who has the parts of generation nearly like another, but at the same time the clitoris grows to a great size, and gives the form of the male penis. This is a very inconvenient disorder, as she is sometimes deprived of the pleasures peculiar to her sex, and suffers much from disorders of the part. From her breasts, and the deficiency of beard, however, she is distinguished from the male; though it frequently and unfortunately happens, that such women are more subject than others to robust and masculine constitutions. It is evident that the sexes here are as completely marked as in other persons, and, to all legal intents and purposes, they are man and woman.

"Some important enquiries may arise upon this subject. As, 1st. How far they are to be considered as impotent. This is, I believe, generally the case, but not always, and must depend upon proof. 2d. Whether they should be permitted to marry? This depends upon the former, but must, I should think, be left to their own choice. 3. Whether change of the sexes might be allowed? This is certainly contradicted in the terms, and will admit of no dispute.

"With regard to the state of life of a child, the following question remains to be decided: At what time may a fœtus be supposed to begin to live? To answer this, we must consider, that conception is made in the ovarium of a female after coition with a male, when the subtile aura of the semen hath so far penetrated into the germen, which may be supposed to contain the outline of the future man, as to produce a turgescence and motion of its circulating humours. At this time, it may be said, that life begins, i. e. immediately after conception. Hence those seem to err: 1st. Who would persuade us, that the fœtus acquires life when it is so particularly active, that the mother becomes sensible of its motions. 2d. Those who think that life does not begin till the seventh or fourteenth day, or even till a month after conception. And, 3d. Those who suppose that a fœtus, as long as it continues in the womb, where it does not breathe, cannot be called a living animal. The whole depends on our ideas of life and animation, and the act of generation to create it. If generation be the cause of animating the rudiments of the future being, and if that animation be construed to be understood by what is meant by life, then it must certainly begin immediately diately after conception, and nothing but the arbitrary forms of human institutions can make it otherwise.

"On this occasion we may enquire, what part of the human body is the seat of animation, or the soul? To which we answer, that evidently it resides most conspicuously in the brain, because that substance being hurt, all the faculties of the soul become disordered; and because all the nerves of the body, which are the great instruments of action, are derived from it as a fountain. But it cannot be supposed that the whole of the brain is the immediate seat of the soul; it is probably confined to what is called the sensorium commune, or a small part from whence the nerves, destined to sense and voluntary motions, draw their origin; as they do likewise from an appendix to it, called the medulla oblongata.

"The next thing to be considered is, what kind of children, when born into the world, are to be deemed endued with life, or have a prospect of living; for a fœtus cannot live out of the womb of its mother.

"1st. Then, no abortion can be said to be endued with life, for if there be some signs of life when it is brought into the world, it cannot continue to live, for it can neither take the aliment which is necessary to its sustenance, nor, if it could take it, can it change such gross food into its tender nature. Some authors have asserted, that children of five or six months have lived; but this is probably a mistake; it being generally agreed, that infants so young cannot sustain the inclemencies to which they must be subject.

"2d. Children of seven months, or one hundred and eighty-two days after marriage, may live, though generally they are puny, and continue but a short time on earth.

"3d. All children above seven months are supposed to be endued with vital principles, and of consequence are allowed the privilege of life.

"The next subject of consideration is that of twins, supposititious births, and superfœtation.

"The right of primogeniture must be determined in natural births, by that which was first born into the world, and which must be decided by the by-standers. If the delivery, however, be made by a passage effected by art, the choice depending on the will of the surgeon, no proper determination can possibly be made.

"In the affair of supposititious births, two questions occur, according as the birth is performed or not. In the former case, a physician may judge, 1st. From those signs in the mother, which distinguish her having been delivered of a child. 2d. From those signs which refer to her incapacity of conception. 3d. From signs of impotency in the father. 4th. From the umbilical cord in the child not appearing as of one just delivered. Some persons look upon the dissimilitude to the parents to be a sign, but this must be very fallacious. Where the supposititious birth depends on the present state of pregnancy, either the proper signs must be examined, or we must wait the event, should those signs deceive us.

"The

"The impregnation of a woman already pregnant, is called a superfœtation. This is either true or false; the former is, when it happens in the womb itself; the latter, when one fœtus is deposited in the womb, the other in the ovarium, the fallopian tube, or the cavity of the abdomen.

"The following requisites are necessary to a superfœtation. 1st. The pregnant woman ought to bear two children, each of a distinct age. 2d. The delivery of these children should be at different times, at a considerable distance from each other. 3d. The woman must be pregnant and a nurse at the same time.

"There have been many doubts about the reality of the superfœtation, but there is no disputing of facts; for which see Graves on Superfœtation, Eisenman's Anatomical Tables, and the Leipsic Memoirs, 1725.

"How this superfœtation is accomplished, is a matter of enquiry, and depends in a great measure on the constitution, or rather the formation of the womb of the mother.

"The last thing to be considered under this head of parturition, is the legitimacy or illegality of births; and this is divided into the time when a child is born after conception, and the conformation of its body. With respect to time, physically considered, (for laws may be as arbitrary as they please in this respect) all abortions, too early births, children of nine months, and those who are late born, even to ten months, may be considered as legitimate in old marriages. Illegitimate with respect to the time of birth, are all perfect and mature children, who are born in the sixth or seventh month after the celebration of marriage; and all late births, when extended to the eleventh, twelfth, or thirteenth month, especially if the husband died of a chronic or lingering disease.

"There are many causes alleged to occasion a delay or prolongation of delivery, such as great care and anxiety; some severe diseases, as violent hæmorrhages, a phthisical disposition, &c. but these, one should imagine, would rather hasten than retard such a circumstance. Experience is the only guide we can follow in such cases, and, for the sake of humanity, the longest time that can be fairly proved, should be the standard to which we should refer.

"With respect to the conformation of the body, all children may be considered as legitimate, who are born at or after seven months; but all abortions are illegitimate. Monsters, likewise, are not to be excluded for any trifling alterations; but where all appearances of human nature are obliterated, it would be wrong to take advantage of such a birth.

"I. Idiotism is, 1st, either born with the subject of it, or appears as soon as the reasoning faculties should begin to expand.

"2d. It is established upon great defects of the memory, and much greater of the judgment, though this is not much attended to.

"3d. Idiots are in general prone to mischief, or to actions over which reason seems to have very little command.

"4th. They have not a proper command over the evacuation of fæces and urine, and drivel at the mouth.

"5th. They have generally strong and hearty constitutions.

"6th. They have a peculiar aspect, which describes a vacancy of thought and inattention to any engagement.

"7th. They have little use of speech, and articulate very incoherently.

"II. Insane persons are either furious or melancholic, both of which acknowledge a great imbecility of the mental faculties, and which are derived from hereditary constitutions, attention of mind, violent passions, the terrors of a false religion, immoderate use of venery, poisons of the narcotic kind, some preceding disorders, the suppression of evacuations, indigestible aliments, a sedentary life, &c. But they differ in the following particulars:

"1st. The furiously insane are naturally of angry and violent dispositions, in the prime of youth, and of a plethoric constitution, and tense fibre.

"2d. They lose all their natural delicacy of manners, and become furious, ungovernable, and are particularly affected by pride, anger, hatred, and revenge, and very often intemperate lust.

"3d. They refuse their food, and yet preserve their strength; they scarcely ever sleep, are continually shifting their ideas from one thing to another, bear the cold with incredible patience, and are not easily affected by medicine.

"4th. They have a peculiar look with their eyes, descriptive of violent anger, mixed with a glariness like that of drunken persons, their eye-lids are constantly vibrating, and their hands, and sometimes the whole body, they keep in motion.

"Melancholy persons are,

"1st. Naturally dull, slowly learning, and easily forgetting, and are sad and melancholy, of a phlegmatic temperament, and relaxed fibre.

"2d. When the disorder seizes them they become abject, fearful, fond of solitude, prone to anger, changeable in their opinions and desires, but fixing their attention upon a single object.

"3d. The belly is constipated, the urine is made in small quantities; the abdomen is distended with wind; a sharp acrid matter is discharged by vomiting; the pulse moves very slowly; the aliment is devoured with greediness; the imagination is perverted so, as that they are persuaded that they are made of glass, china, &c. and lastly, and worst of all, they are induced to put a period to their existence.

"4th. Their eyes have a dull, heavy, and stupid look; they seldom move, but continue in one posture a very long time."

---

*Observations on those Diseases of Females which are attended by Discharges;* by CHARLES MANSFIELD CLARKE, Member of the Royal College of Surgeons, &c. Longman and Co. 8vo. pp. 304.

(Concluded from p. 247.)

CARCINOMA UTERI.—Dr. Baillie, in his Morbid Anatomy, describes three states of the womb which have been discovered on dissection:

dissection: scirrhus enlargement, which seldom if ever ulcerates; the fleshy tubercle, and malignant ulcer of the uterus, which always begins at the cervix, and which is very fatal. The tumor which arises from the glandular cervix and the thickening of the cervix, are alone considered by Mr. Clarke to be cancer, and both are disposed to ulcerate.

Our author, with suitable modesty, gives Dr. Baillie the credit of first distinguishing the malignant ulcer of this part from cancer. But we must observe, that the merit is not due to Dr. Baillie, but his predecessors. The first edition of Morbid Anatomy confounded the two diseases. The subsequent ones not only corrected this error, but, with a candour worthy of the author, accompanied the correction with the following note: "This diseased change I formerly confounded with the scirrhus enlargement of the uterus, considering them as varieties of the same disease, and therefore blended their description together; but in consequence of the accurate observations of Dr. Adams, in his Essay on Morbid Poisons, I have thought it proper to separate them."—See Baillie's Morbid Anatomy, third edition, page 364, note.

We shall therefore direct our readers to the first edition of Adams on Morbid Poisons: here, we believe, for the first time, is to be found the distinction made by a modern author. But Dr. Adams does not assume the credit to himself. He acknowledges that he derived the information from Mr. Hunter's lectures and conversations; and draws a confirmation of its truth from a writer whose opinions must have been unknown to Mr. Hunter, on account of the language in which he wrote, inasmuch as he has not been attended to by any modern author.

"There is an ulceration of the os uteri of a distinct kind from that just mentioned, although equally fatal. This will be described in a future part of this work, under the head of purulent discharges, by the name of the corroding ulcer of the os uteri.

"The cases described by Dr. Baillie under the title of Scirrhus Uteri and Tubercle of the Uterus, the author means to consider together, under that of the Fleshy Tubercle of the Uterus; for the uterus in both has tubercles, either arising from its surface externally or internally, or imbedded in its substance. In both, few except mechanical symptoms, are present: in neither does ulceration take place. In both, the tubercles are found at a distance from the cervix of the uterus, and both sometimes continue for many years without producing much inconvenience."

It is essential to remark, that mere induration and enlargement of the body of the womb are insufficient to characterise cancer of that viscus. The seat of this disease is mostly, *if not always*, in the cervix.* "On

* "Etsi cancer etiam ipsi uteri substantiæ accidere potest, tamen hoc rarius accidit, et vix tum satis cognoscitur, multo minus curatur; frequenter vero in cervice uteri generatur, quapropter hoc loco

"On examination this part will be found thickened, and, with a resisting feel resembling that of gristle, or a distinct tumour, will be perceived arising from some part of the cervix uteri, the other parts remaining healthy. In either case, pressure upon the diseased parts produces pain of a lancinating kind.

"The os uteri will be found also to have undergone a change. It becomes larger than natural, still however retaining its original shape. This open or gaping state of the os uteri sometimes is sufficient to admit the extremity of a finger, which, when introduced into it, feels as if surrounded by a firm ring. The parts will sometimes have undergone all the changes of structure above related when no local symptoms have been apparent, and when the disease has only been ascertained by an examination, suggested by the failure of remedies in relieving the supposed disease of the stomach or kidney."

"When carcinomatous tumours are cut through with a knife, they offer a good deal of resistance, and appear sometimes as hard as cartilage. The cut surface presents an appearance of white lines, which run pretty regularly with regard to each other, but the directions of which vary according to the shape of the tumour."*

The fleshy tubercle of the uterus is said to be situated in the body of the uterus, and never ulcerates.

"Two varieties of cancer are observed in early stages:

"1. There is a firm tumour, of a rounded form, springing from the surface of the cervix uteri, or imbedded in it, whilst the other parts of the uterus are perfectly healthy, except that its parieties are thickened as the disease advances, and that its cavity becomes larger than that of an healthy unimpregnated uterus.

"2. Instead of any distinct tumour, the whole of the cervix of the uterus becomes larger and harder; and, if this thickened part is examined by cutting into it, it puts on the same appearance which a regular carcinomatous tumour possesses.

"The two cases proceed differently. In addition to the usual symptoms of carcinoma, there will sometimes be found in the first variety of the disease some mechanical symptoms, depending on the pressure made by the tumour upon the neighbouring parts; which symptoms will be more or less severe, according to the size and situation of the tumour itself.

"In the second variety of the disease these symptoms do not exist; because the carcinomatous thickening of the cervix uteri rarely acquires a sufficient size to produce them.

"There is reason to believe that carcinomatous tumours make a very rapid progress when any violence has been done to them, as the following case will show. A woman, about the age of forty, fell into labour: the head of the child presented; and, little progress

---

de eo agemus: Isque nunc est sine ulcere, nunc exulceratus."—Sennertus, lib. iv. de Morbis Mulierum, cap. 11.

* "Vide Dr. Baillie's Morbid Anatomy."

being being made, a consultation was thought necessary, and the author was desired to see her. Upon examination, the os uteri was found dilated to the size of a half-crown, the cervix of the uterus was greatly thickened in every part, and felt like cartilage; it was also very tender. Upon inquiry, it appeared that the woman had been liable, during the latter part of her pregnancy, to a profuse discharge of mucus, and to occasional attacks of pain in the lower part of the abdomen. Two days elapsed before the os uteri was completely dilated, and the dilatation was performed with greater pain than usual. The head of the child at length passed through it. After the labour, the pain and discharge were greatly increased, and the woman died in a few days. Upon examining the cavity of the abdomen after death, the body of the uterus was found contracted as much as it generally is at the same period of time after delivery; but the cervix was very much thickened, and had begun to ulcerate. The parts are preserved in the collection of the author. It is to be presumed, that the disease formed after the commencement of the pregnancy, and that it became more active in consequence of the violence done to it in labour. Comparing this case with others in the progress of which occasional examinations are made, it is probable that it must have proceeded with great rapidity.

" A mucous discharge from the vagina is a very constant attendant upon the complaint; and, by the local evacuation, it in some measure checks the progress of it.

" This mucous discharge is sometimes tinged with blood, and particularly when the patient indulges in eating and drinking, or where the food taken has been of a stimulating quality. If the woman uses much exercise, pure blood sometimes comes away, and in such large quantity as to produce great weakness, and occasionally fainting. Generally whilst there are discharges of blood in moderate quantity, the tumour remains almost stationary, increasing little in size, and producing little or no uneasiness. The author has seen many instances of women with a diseased uterus attended by distressing symptoms, who, after having been attacked by large bloody discharges, so as to make them faint, in any other than the horizontal posture, and to bring on general anasarca, have continued free from every symptom of the specific disease for many months. In some instances where the woman has died, it has been from weakness and the dropsical symptoms, and not from the symptoms belonging to the original disease. This is the reason why many cases of menorrhagia ending in dropsy are unmanageable; because they depend upon organic disease of the uterus, which is never perhaps known, or, if known, baffles the art of medicine.

" In carcinoma uteri, if menstruation has not ceased, it becomes for the most part irregular, and is more profuse than it ought to be.

" The mechanical symptoms produced by tumours in the pelvis sometimes attend carcinoma; but the patient seldom suffers much from them, since the size of the carcinomatous tumour is not often great enough to cause them. Œdema of the lower extremities sometimes attends; but this is not generally the effect of pressure

 upon upon the trunks of the absorbents; for it does not appear until the frame has been much weakened by the long continuance of the disease, and the disposition to anasarca is general.

"Difficulty of passing urine rarely occurs in this complaint; but strangury, arising from the consent between the uterus and the bladder, is seldom wanting. In some instances it accompanies the disease from the beginning, but in others it ushers in the symptoms which immediately precede the conversion of the disease into the state of ulceration.

"The inner membrane of the bladder is found to secrete, in some cases, a larger quantity of transparent mucus, which comes away with the urine, and falls to the bottom of the urinal.

"The seat and the course of the pain, resembling the passage of a calculus from the kidney to the bladder, have led to a mistaken idea of the disease; and in some instances the false opinion has been strengthened by another symptom which attends both diseases; namely, urticaria or nettlerash.

The treatment must be regulated according to circumstances. Our author has not forgotten the use of local blood-letting in cancerous affections, which, in this case, is better effected in the loins by cupping or leeches. The necessity of frequent ablution of the vagina with tepid water, mild aperients, and abstemiousness of diet, seems to be obvious. Urticaria, in this case, is considered as arising from the state of the stomach: the mode of relief must, therefore, consist in regulating the secretions of that viscus. Alkalies, with aromatic bitters, are the best remedies for this purpose. Preparations of iron have been recommended in cancerous complaints; but in those cases which have fallen under the care of the author, they have done more harm than good.

"POLYPUS UTERI.—The true character of this disease can only be ascertained by examination. This will discover an insensible tumour projecting through the os uteri, by which its neck is entirely encircled, so that the finger can be completely passed round it.

"The only diseases which can be mistaken for polypus are an inverted uterus, and the cauliflower excrescence of the os uteri. The history of the case from its commencement, and the insensibility of polypus, will distinguish it from the first: besides which, unless the uterus is only partially inverted, (a very rare occurrence,) the tumour will not be encircled by the os uteri. The irregularity of the surface of the cauliflower excrescence, the circumstance of its originating from the substance of the os uteri, with a broad base, and not coming through it, and the watery discharge which attends this disease, will prevent the practitioner from confounding it with polypus.

"There is, however, a tumour which has been looked upon and treated as polypus, which ought to be distinguished from it, on account of the prognostic to be given respecting its termination, and also because it does not admit of the same successful mode of treatment as polypus.

"This

"This disease consists of a tumour, which is insensible, which has an unequal ragged surface, which comes down from the cavity of the uterus into the vagina, surrounded by the os uteri, and without a narrow neck."

"The tumour which resembles polypus has been described by Levret and by Herbiniaux by the name of Vivace, although admitting of removal by the ligature, is disposed to return; other newly-formed irregular portions shooting down into the vagina, and this with a rapidity of growth not belonging to polypus."

There is, however, no objection to the ligature, but the success of the operation will be questionable. A mode of applying the ligature to these tumours may be found at the commencement of the present Number, which, in our opinion, is infinitely superior to any before employed.

"The FLESHY TUBERCLE is a hard whitish tumour, sometimes nearly as firm as cartilage, situated sometimes upon the surface of the uterus, between the muscular and the peritonæal coat, sometimes projecting into the cavity of the uterus, and occasionally imbedded in its substance.

"When it projects into the cavity of the uterus, its surface is smooth: the contrary is generally the case when it forms upon the outer surface of the uterus, the tumour having a granulated appearance. These tumours are sometimes not much larger than a pea; sometimes they weigh several pounds, and occupy a great part of the cavity of the abdomen.

"In general, when the tumour is large, the texture is less firm than when it is of a smaller size. It appears to be composed of distinct parts, connected by a close cellular membrane, the diseased tumour itself being opaque, and the connecting membrane more or less transparent. If coloured injection be thrown into the vessels of the uterus, so as to make the substance of the uterus quite red, none of it passes to the tumour of fleshy tubercle. In the collection of Mr. Abernethy, assistant-surgeon to St. Bartholomew's Hospital, there is a very good preparation showing this fact."

These tumours are situated at a distance from the cervix uteri, and have sometimes been mistaken for hydrops ovarii, and pregnancy. They neither suppurate nor ulcerate: the surrounding parts may, but the tubercle remains the same.

In the 18th and 19th Chapters, the author describes warts, and the vascular tumour of the orifice of the meatus urinarius, upon neither of which we feel it necessary to detain our readers.

"*A Thickening of the Cellular Membrane surrounding the Urethra throughout its whole extent, accompanied by a varicose State of the Vessels of the Part.*—On examination, a bulbous tumour will be found situated behind the pubis; and if much pressure is made upon it, pain will be produced, but not of a severe kind.

"A mucous discharge always attends this disease, secreted probably in part by the membrane of the urethra, and in part by that which lines the vagina.

"If

"If the parts are exposed, and the patient presses down, the diseased part will be brought into view, putting on the appearance of a tumour, but which is nothing else than a thickening of the urinary passage. On the surface of this thickened part blood-vessels ramify, of a size large enough to admit of being opened by the point of a lancet.

"When the patient is in an erect posture, the size of these vessels increases, and she complains of a sense of fulness in the parts: when she lies down, the vessels carry less blood, and the sensation of fulness is diminished. If pressure is made upon the part, the swelling and redness subside for a time, but both return directly upon the pressure being discontinued. Sometimes a pouch forms in the posterior part of the urethra, in which a few drops of urine lodge, and from which situation it may be pressed out by a finger applied to the part. If a catheter is introduced into the urethra, it may be carried backwards to the part where this lodgement of urine is found. Upon this cause depends, perhaps, one of the most troublesome symptoms of the disease,—a frequent desire to make water, both in the night and during the day, so as to interfere with the patient's rest.

"The disease seems to consist of an enlargement of the blood-vessels of the part; because, when the vessels are emptied of their contents, the size of the tumour diminishes. Judging from the colour of the tumour, there is reason to believe that the enlarged vessels are principally veins.

"The most speedy and effectual mode of relieving the patient is by emptying the vessels, either by puncturing them with a lancet, or by the application of leeches: either may be employed, according to circumstances. The size of the vessels and of the whole tumour will be diminished by these means, and its colour will be changed from a deep red to the proper colour of the part.

"The fulness of the vessels being removed, lotions composed of solutions of lead may be applied cold to the parts, and these should be changed as often as they become warm. After a day or two, weak solutions of muriate of ammonia or of sulphate of zinc may be used: at first, the openings made by the leeches or the lancet would be inflamed by them. Pressure is serviceable, and may be applied either by introducing a piece of wax candle, or a small roll of linen which may be previously dipped in the lotion."

The two remaining chapters are on "*the transparent Mucous Discharge from the Vagina, not accompanied by any alteration of Structure of the Sexual Organs*," and the "*Case of transparent Mucous Discharge depending on Debility;*" but which, as they are more generally known, and their treatment understood, it is unnecessary for us to notice. We cannot take leave of this interesting performance without expressing our sincere hope that the promised continuation of the subject may not long be withheld from the public.

MEDICAL

